# Ranking Decision-Making Criteria for Early Adoption of Innovative Surgical Technologies

**DOI:** 10.1001/jamanetworkopen.2023.43703

**Published:** 2023-11-16

**Authors:** Haitham Shoman, Nisha D. Almeida, Michael Tanzer

**Affiliations:** 1 Department of Experimental Surgery, Faculty of Medicine, McGill University, Montreal, Quebec, Canada; 2 Vanier Scholar, Canadian Institutes of Health Research; 3 Health Technology Assessment Unit, McGill University Health Centre, Montreal, Quebec, Canada; 4 Division of Clinical Epidemiology, McGill University, Montreal, Quebec, Canada; 5 Division of Orthopaedic Surgery, McGill University, Montreal, Quebec, Canada

## Abstract

**Importance:**

There is no decision-making framework in the early-adoption stage of novel surgical technologies, putting the quality of health care and resource allocation of the health care system at risk.

**Objective:**

To investigate relevant weighted criteria that decision-makers may use to make an informed decision for the early adoption of innovative surgical technologies.

**Design, Setting, and Participants:**

This multi-institutional decision analytical modeling study used a mixed-methods multicriteria decision analysis (MCDA) and had 2 phases. First, a panel of 12 experts validated decision criteria in the literature and identified additional criteria. Second, 33 Canadian experts prioritized the main criteria (domains) using the composition pairwise-comparison weight-elicitation method (analytical hierarchy process model) and ranked their subcriteria using the direct-ranking elicitation method (Likert scale). Data were analyzed, and response consistency was estimated using the consistency ratio. Analysis of variance was used to assess for significant differences between expert responses. The MCDA was conducted at McGill University between 2021 and 2023. Data were collected nationally by inviting experts in Canada.

**Main Outcome and Measure:**

Criteria domain weights and subcriteria rankings. Priority vectors, which are priority scores analyzed and prioritized from expert responses, were used to rank criteria domains and subcriteria for decision-making on adopting new innovative surgical technologies.

**Results:**

A total of 45 experts (33 male [73.3%] and 12 female [26.7%]) were invited with different levels of education (22 experts with MD or equivalent, 13 experts with master’s degree, and 12 experts with PhD degree) and years of experience (4 experts with <10, 12 experts with 11-20, 18 experts with 21-30, and 11 experts with >30 years). Surgeon experts (23 individuals) were from all surgical disciplines, and nonsurgeon experts (22 individuals) were administrative officers in surgical device procurement, health technology assessment experts, and hospital directors. A total of 7 domains and 44 subcriteria were identified. The MCDA model found that clinical outcomes had the highest priority vector, at 0.429, followed by patients and public relevance (0.135). Hospital-specific criteria (priority vector, 0.099), technology-specific criteria (priority vector, 0.092), and physician-specific criteria (priority vector, 0.087) were the next most highly ranked. The lowest priority vectors were for economic criteria, at 0.083, and finally policies and procedures, at 0.075. There was consensus in the responses (consistency ratio = 0.006), and there were no statistically significant differences between expert responses.

**Conclusions and relevance:**

This study weighted priority criteria domains in importance and established ranked subcriteria for decision-making of early adoption of surgical technologies. Putting these criteria into a framework may help surgeons and decision-makers make informed decisions for the early adoption of new surgical technologies.

## Key Points

**Question:**

What are the relevant weighted criteria that decision-makers can use to make an informed decision for the early adoption of innovative surgical technologies?

**Findings:**

In this decision analytical modeling study, clinical outcomes were the most important criteria to guide surgeons and nonsurgeons in the early adoption of new surgical technologies. Responses from surgeons and nonsurgeons were not statistically significantly different, suggesting a similar priority-setting criteria among all stakeholders.

**Meaning:**

These findings suggest multiple prioritized and weighted criteria, which may be indispensable in making informed decisions in the early adoption of new surgical technologies to judiciously use financial resources and maximize patient outcomes.

## Teaser

This decision analytical modeling study investigates the weighting of criteria used by surgeons, surgical administrators, and health technology assessment experts to make decisions concerning the early adoption of innovative surgical technologies.

## Introduction

Continuous innovation in surgery is crucial in improving patient outcomes, minimizing complications, and allowing more effective and efficient surgical care.^1^ The process of technology adoption over time is typically illustrated as a classical bell curve distribution, with the first group of people to use a new product called innovators, followed by early adopters, the early majority, and the late majority; the last group consists of laggards.^2^ While surgeons are generally risk averse and favor the status quo, there are surgeons who are early adopters of new technologies.^3^ These surgeons are more likely to be opinion leaders, closely watch for new innovations, embrace technological innovation, and be ready to adopt new technologies early.^4^

The decision-making process to purchase and adopt new surgical technology is complex and multifaceted, involving multiple stakeholders. It is not standardized and varies by country and health care system. In the US, after a technology is approved by the Food and Drug Administration, technology adoption is decided by the hospital administration and surgical departments.^5,6^ In the United Kingdom, after a new technology receives approval by the Medicines and Healthcare Regulatory Authority,^6^ the National Institute for Health and Care Excellence conducts health technology assessments (HTAs) and provides recommendations for technology adoption to the National Health Service based on a cost-effectiveness analysis.^7^ In Canada, the decision to adopt and reimburse medical devices is decentralized in the publicly funded health care system.^8^ The Health Canada Medical Devices Directorate regulates medical devices for human use.^9^ While HTA is not mandatory in Canada, the Canadian Agency for Drugs and Technologies in Health provides information to some provincial HTAs, while other provinces have their own provincial HTA agencies and bodies, such as in Quebec (Institut National d’Excellence en Santé et en Services Sociaux) and Ontario (Health Quality Ontario, Evidence Development and Standards Division).^10^ In these provinces, technologies are assessed based on requests from hospital administrators and physicians.^10^

By using evidence-based criteria, decision-makers can evaluate the potential impact of a new surgical technology, weigh disadvantages, and make an informed decision that aligns with organization goals, needs, and resources. Although cost, clinical safety, and effectiveness are frequently considered among the most important criteria, numerous other criteria influence the decision-making process.^11,12,13^ These include criteria specifically related to the technology, surgeon, patients being treated, hospital, and health care system.^14,15^ For example, in the highly competitive US health care system, 1 important criterion is the potential benefit for an institution to promote itself as cutting edge and a center of excellence and, thus, accept a higher volume of patients.^16^ In Canada, other criteria include disease burden, equity of access, and feasibility of implementation.^10^

The weighting of criteria is an essential step in decision-making because it simplifies the process by prioritizing the most relevant factors and allows for a more objective decision that aligns with organization goals and priorities. Organizations may have their own unique criteria that are specific to their needs and circumstances. Currently, there is no decision-making framework for the early-adoption stage of novel surgical technologies. The aim of this study was to investigate the relevant weighted criteria that decision-makers, including surgeons, HTA experts, and surgical administrators, may use to make informed decisions for the early adoption of innovative surgical technologies.

## Methods

No ethical or institutional review board approval was needed for this decision analytical modeling study because no medical devices, drugs, or patients were involved in the study; the McGill University Health Centre institutional review board does not require approval for public health surveillance activities, as defined by the Quebec Ministry as evaluation of care and services. Participants provided consent to take part in the study. The Consolidated Health Economic Evaluation Reporting Standards (CHEERS) reporting guideline was adopted and modified to fit this multi-criteria decision analysis (MCDA) study by replacing the time horizon and economic aspect with using the analytical hierarchy process (AHP) and weighting of criteria as per Professional Society for Health Economics and Outcomes Research (ISPOR) guidance.

### Study Design and Setting

Surgical department chairs, divisional chiefs, and procurement directors from the major Canadian university teaching hospitals, as well as members of all Canadian provincial HTA committees, were identified from their respective websites. Each was sent an invitation by email explaining the project and requesting participation in the study. An attempt was made to recruit individuals from all provinces and from diverse surgical disciplines. In the first round of surveys, 12 experts were questioned only to confirm criteria in the published literature or provide additional criteria. Experts were from McGill University and all divisions of surgery and included directors of all departments responsible for procurement at the McGill University teaching hospital. The MCDA study then used these literature-based criteria and the few additional criteria identified by experts. Decision criteria were prioritized and ranked using MCDA in accordance with the ISPOR.^17^ MCDA considers multidimensional factors and enables comparison in 1 overall appraisal. Participants surveyed for this study included 3 main stakeholder groups: surgeons, hospital administrators involved in the decision-making regarding and procurement of surgical devices, and provincial HTA committee members. All participants were experts in their fields, worked in Canada, and were identified by study investigators and by contacting HTA agencies across Canada (Table 1). After experts consented to participate in the study through email invitations, they were sent questionnaires electronically, and responses were collected anonymously.

**Table 1.** Participant Demographics

Characteristic	Respondents, No. (%)			
	Overall (N = 45)	Surgeons (n = 23)	HTA experts (n = 13)	Other (n = 9)
Sex				
Male	33 (73.3)	20 (87.0)	7 (53.9)	6 (66.6)
Female	12 (26.7)	3 (13.0)	6 (46.2)	3 (33.3)
Highest level of education				
MD or bachelor’s degree^a^	22 (48.9)	15 (65.2)	2 (15.4)	5 (55.6)
Master’s degree	13 (28.9)	4 (17.4)	4 (30.8)	5 (55.6)
PhD	12 (26.7)	6 (26.1)	6 (46.2)	0
Years of experience				
<10	4 (8.9)	1 (4.3)	3 (23.1)	0
11-20	12 (26.7)	2 (8.7)	5 (38.5)	5 (55.6)
21-30	18 (40.0)	12 (52.2)	2 (15.4)	4 (44.4)
>30	11 (24.0)	8 (34.8)	2 (15.4)	1 (11.1)

Abbreviation: HTA, health technology assessment.

^a^ Bachelor’s degree could include BSc, BA, MBBS, and MBBcH.

### Selection of Decision-Making Criteria Categories

We identified 33 decision-making subcriteria within 7 domains from a previously published study.^18^ In the first round of surveys, 12 experts were sent a questionnaire to evaluate the pertinence of these 33 subcriteria and determine if further subcriteria needed to be added. All newly identified subcriteria were then added to 1 of the 7 domains with the corresponding theme. Final decision-making domains and subcriteria were evaluated in the second step of the analysis. These experts were not part of the next round of surveys.

### Prioritization and Ranking of Domains

In the second round of surveys, 33 of 45 experts agreed to participate in the study (response rate, 73.3%). Experts included 16 surgeons, 6 hospital administrators, and 11 provincial HTA committee members. Experts were sent a questionnaire that had 2 main objectives: first, to rank the importance of each subcriteria and second, to determine which domain was more important. We determined that the meaning of each domain in the questionnaire was clear to experts because each criterion was listed with its subcriteria, no respondents expressed uncertainty with a criterion, and no experts left a criterion question unanswered because they were not clear on its meaning.

### Data Analysis and Model Development

Data were analyzed using a mixed-model methodology that includes domain prioritization by weighting using the AHP model and subcriteria ranking using the direct elicitation method, the Likert scale. To obtain an importance ranking, we asked respondents to consider each subcriterion independently and rank them on a 5-point Likert scale, with 1 characterizing an irrelevant criterion and 5 characterizing an absolutely relevant criterion (eAppendix in Supplement 1). Responses from the 33 experts were then analyzed using the arithmetic mean, and subcriteria were reordered based on their rankings from most to least important.

Second, domains were prioritized using the compositional pairwise comparison matrix, the AHP model (eAppendix in Supplement 1). The AHP calibrates subjective and objective aspects of a decision through 5 steps: hierarchical structure development pairwise comparison, criteria-weight calculation, computation of option scores matrix, and ranking of criteria.^19,20,21^ A pairwise comparison was used to determine which criteria were more important by comparing 2 domains at a time using a 9-point Saaty scale, with 1 indicating equal importance and 9 indicating absolute importance of 1 criterion over the other.^19,20,21^ The geometric mean was then estimated to combine individual pairwise-comparison judgments of the 33 experts into a pairwise-comparison matrix. The AHP model used results of the pairwise-comparison matrix and normalized scores to derive the numerical weights priority vector for each domain, allowing the 7 domains to be compared in a consistent and rational approach (eTable 1 in Supplement 1).^19,20,21^

### Statistical Analysis

To verify the consistency of comparisons, the consistency index was applied for the entire pairwise-comparison matrix.^20,21^ The consistency ratio was then used to determine whether the matrix was sufficiently consistent with a random index (equaling 1.32) for the 7 categories.^20,21^ A consistency ratio less than 0.10 indicated that there was consistency within expert responses and the matrix was sufficient (eTable 2 in Supplement 1).

A Microsoft Excel-Office 365 version 2309 (Microsoft Corp) package was used to develop the MCDA model and statistical analysis. A 1-way analysis of variance (ANOVA) was performed to test for statistically significant differences between the priority vectors of domains and subcriteria ranked by surgeons only, nonsurgeons only, and all experts. A *P* value < 0.05 was considered statistically significant.

## Results

A total of 45 experts (33 male [73.3%] and 12 female [26.7%]) were invited for domain and subcriteria validation, including 23 surgeons and 22 nonsurgeons (13 HTA experts, and 9 administrators) (Table 1). There were 11 experts with more than 30 years of experience and 4 experts with less than 10 years of experience; 12 experts had 11 to 20 and 18 experts had 21 to 30 years of experience. There were 22 experts with an MD or equivalent, 13 experts with a master’s degree, and 12 experts with a PhD degree. In the first questionnaire, experts validated and confirmed that the 7 domains and 33 subcriteria were pertinent. In addition, experts identified 11 new subcriteria that were important and were grouped with the 33 subcriteria identified in the literature.^18^ These additional subcriteria were cost-effectiveness, depreciation cost, certification of technology, percentage of use, maintenance availability, availability of evidence, alternatives available, being an academic center of excellence, evidence of peer-reviewed publications, and disease burden.

### Analysis of Domains

**Figure 1.**
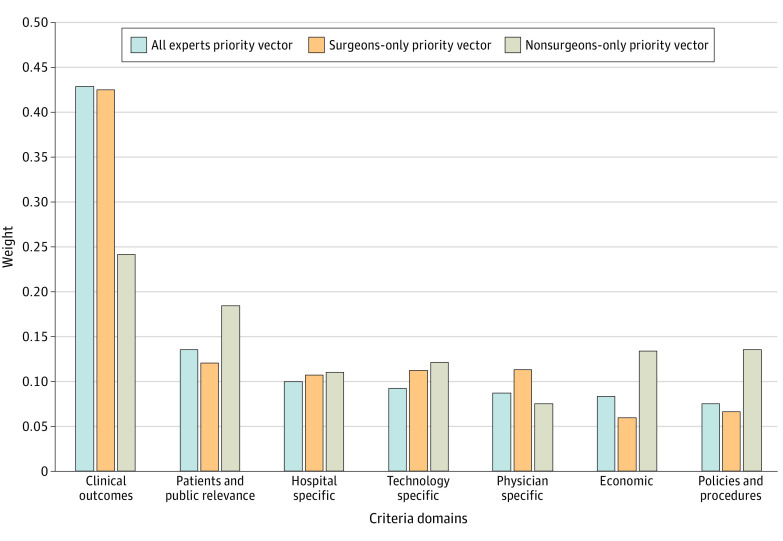
Priority Weights of 7 Main Criteria Domains

Table 2 shows the full model and analysis results. Of 7 domains, clinical outcome was determined to be the most important domain, accounting for 42.9% (priority vector, 0.429) of the decision-making process. This domain included subcriteria of safety, efficacy, effectiveness, prevention of adverse effects, evidence of peer-reviewed assessments, and disease burden. This was 3 times more important than the next most important domain, patients and public relevance (priority vector, 0.135). Remaining domains each contributed less than 10% (Figure 1). Priority vectors were 0.099 for hospital-specific criteria, 0.092 for technology-specific criteria, 0.087 for physician-specific criteria, 0.083 for economic criteria, and 0.075 for policies and procedures.

**Table 2.** Representation of Criteria Prioritization for Surgical Technology Adoption^a^

Criterion and subcriteria	Priority vector score^b^
Clinical outcomes	0.429
Safety	4.939
Effectiveness	4.909
Efficacy	4.758
Prevention of adverse effects	4.545
Evidence of peer reviewed publications	4.364
Disease burden	4.030
Patients and public relevance	0.135
Human responses and patient experience	4.364
Population health impact	4.242
Access	4.061
Social and demographic	3.424
Publicity and awareness	3.152
Hospital specific	0.099
Feasibility of implementation	4.606
Relevance	4.545
Strategic fit	4.364
Standards of care	4.333
Being an academic center of excellence	4.281
Structural and management support	4.121
Service coordination	3.879
Sense of security	4.121
Availability of the technology	4.061
Training	4.030
Percentage of use	3.939
Innovation champions	3.879
Flexibility of usage	3.727
Technology specific	0.092
Availability of evidence	4.455
Quality	4.424
Efficiency	4.313
Maintenance availability	4.273
Innovation	3.879
Alternatives available	3.688
Technology simplicity	3.576
Real-time feedback	3.515
Physician specific	0.087
Sense of security	4.121
Availability of the technology	4.061
Training	4.030
Percentage of use	3.939
Innovation champions	3.879
Flexibility of usage	3.727
Economic	0.083
Cost-effectiveness	4.290
Economic impact	4.031
Cost	3.781
Depreciation cost	2.774
Policies and procedures	0.075
Ethical	4.273
Certification of technology (Health Canada–ISO)	4.156
Sustainability	3.909
Environmental	3.636
Legislative	3.455
Enforcement	3.129
Appeals	2.818
Political	2.667

Abbreviation: ISO, International Organization for Standardization.

^a^ Prioritization is from the analytical hierarchy process model.

^b^ Domain prioritization by weighting used the analytical hierarchy process model, and subcriteria ranking used the direct elicitation method, the Likert scale.

In a subanalysis for surgeons only, the clinical outcomes domain was again found to have the highest priority vector, at 0.425. This was again followed by patients and public relevance, with a priority vector of 0.12. Next, was physician-specific criteria (priority vector, 0.113), technology-specific criteria, (priority vector, 0.112), and hospital-specific criteria (priority vector, 0.107). The lowest priority vectors were again found with policies and procedures (0.066) and finally economic domains (0.059).

In a subanalysis including nonsurgeons only, the clinical outcomes domain was again found to have the highest priority vector, at 0.242, followed by patients and public relevance, with a priority vector of 0.184. This was followed by policies and procedures (priority vector, 0.135), economic-specific criteria (priority vector, 0.133), and technology-specific criteria (priority vector, 0.121) domains. The lowest priority vectors were hospital-specific criteria (0.110) and finally physician-specific criteria (0.075). There were no statistically significant differences in priority weights in the 7 domains from the 3 groups.

### Subcriteria Analysis

**Figure 2.**
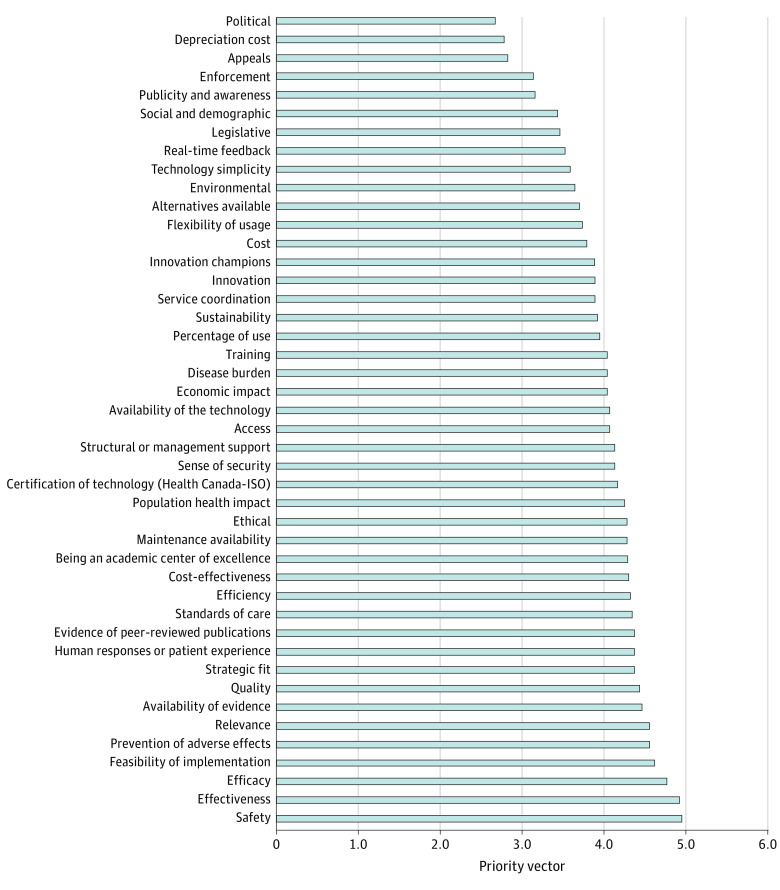
Prioritization of 44 Subcriteria by Rank Subcriteria were prioritized from responses of all experts by their ranks. ISO indicates International Organization for Standardization.

**Figure 3.**
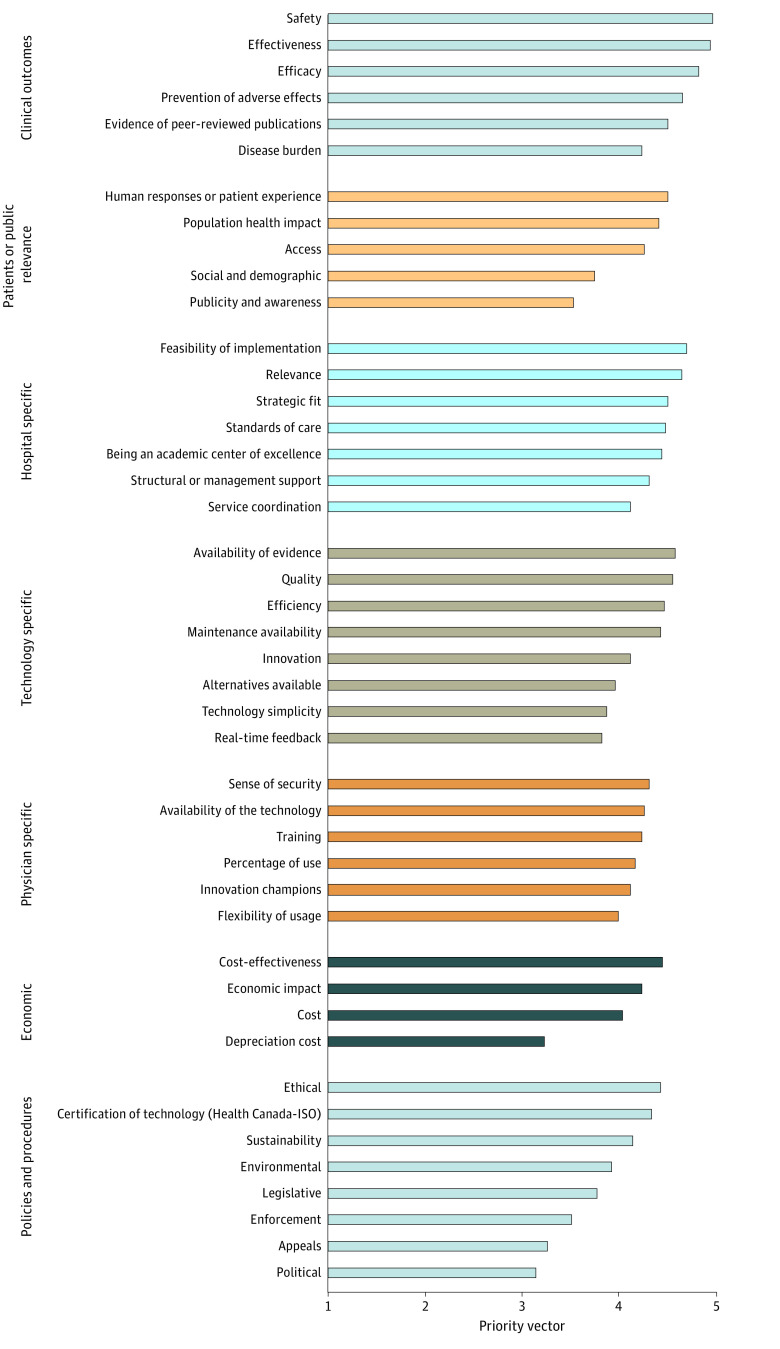
Prioritization of 44 Subcriteria by Criteria Domain Subcriteria were prioritized according to their criteria domains by all experts. ISO indicates International Organization for Standardization.

Overall, safety was ranked as the most important of 44 subcriteria, with a priority vector score of 4.939, followed by effectiveness (priority vector, 4.909), efficacy (priority vector, 4.758), and feasibility of implementation (priority vector, 4.605). The lowest-scoring subcriteria were appeals (priority vector, 2.818), depreciation cost (priority vector, 2.774), and political impact (priority vector, 2.667) (Figure 2 and Figure 3).

The subanalysis of surgeons only identified safety as the most important subcriterion (priority vector, 4.941), followed by effectiveness (priority vector, 4.882), being an academic center of excellence (priority vector, 4.813), and efficacy (priority vector, 4.706). The lowest-scoring subcriteria were appeals (priority vector, 2.941), depreciation cost (priority vector, 2.688), and political impact (priority vector, 2.588).

The subanalysis for non-surgeons identified that safety and effectiveness were equally the most important subcriteria (priority vector, 4.938), followed by efficacy, feasibility of implementation, and structural management equally at a priority vector of 4.813. The lowest-scoring subcriteria were political (priority vector, 2.75) and publicity and awareness and appeals (priority vector, 2.688).

The consistency ratio of comparisons was found to be 0.006 (<0.1), indicating that there was consistency within expert responses and that the matrix was sufficient. In addition, the statistical analysis using ANOVA showed that there was no significant difference in priority vector weights among responses from all expert groups, suggesting that priority vectors from all expert responses may be used as weights to guide adoption decisions for surgeons and decision-makers. There were no significant differences in responses among experts by demographic factor.

## Discussion

This decision analytical modeling study identified clinical outcomes as the most important domain in decision-making regarding early adoption of innovative surgical technologies, accounting for 42.9% of the decision-making process. Safety, effectiveness, and efficacy were the most important individual subcriteria.

This study’s finding that clinical outcomes were of principal importance aligns with the rationale of adopting technologies to improve patient care. Patient safety and effectiveness were considered by surgeons and nonsurgeons to be the most important factors in adopting new technologies^13,14,15,16,17,18,19,20,21,22,23^ given that clinical outcomes are the mainstay for determining health-related quality of life and life expectancy.^22,24^ However, previous studies did not consider the technology adoption life cycle. In the early-adoption phase, the extent of clinical outcomes information is limited and may comprise only a few publications and conference presentations. This does not negate the importance of clinical outcomes but suggests that the expectation of having definitive scientific confirmation is unrealizable, clinical outcome data will be short term in nature, and other sources of data need to be used. These data include all available data from premarket and postmarket phases, such as postmarket surveillance reports, registries containing unpublished clinical performance or effectiveness data, adverse events held by the manufacturer or regulatory authorities, and any clinically relevant field corrective actions, such as recalls, notifications, and hazard alerts. Furthermore, when assessing a new surgical technology, it is critical that the current standard of care be considered and used as the standard of comparison. In addition, given the paucity of data, the possible adoption of the new technology needs to be considered in the context of the currently available technology.

Results from this study have several policy implications. First, the agreement between surgeons and nonsurgeons suggests that the decision-making criteria can be harmonized. Surgeons are the primary users of surgical technologies and have a deep understanding of the technical aspects of the procedure and needs of the patient. Nonsurgeons, such as administrators, have a broader perspective on the financial and organizational aspects of the technology adoption.^25^ Agreement by these groups suggests that the criteria may be relevant and appropriate and that they consider the different perspectives and expertise of both groups.^4,11,12,14^

Second, our results suggest that HTA bodies should include representation of all stakeholders, including surgeons. Surgeons play a critical role in the identification of new technology in the early-adoption phase given that clinical results may be primarily from conferences and individual communications rather than extensive published literature and so would be available later in the technology life cycle.^25^ This information can provide important insight into the indications and benefits of new technologies.^13,25,26^

Third, this study’s findings suggest that not all criteria are or should be equally weighted in importance. Not all criteria were considered as critical given that they may have had different levels of impact on patient outcomes and the overall success of the technology. By assigning different weights to criteria, decision-makers can ensure that the most important factors are given the most consideration when making decisions about new surgical technology adoption. The importance of weighing each criterion is particularly relevant given that the cost of the technology is commonly felt to be a primary criterion in decision-making.^16,23,27^

Fourth, this study found that economic and policies and procedures domains were the least important and cost of the technology ranked 32 of 44 among subcriteria. The cost of technology should be considered in the long term rather than just considering upfront costs. A technology that may have a higher upfront cost but that is associated with better patient outcomes and fewer complications in the long term may ultimately be more cost-effective. Cost-effectiveness analyses tend to use endogenous costs of technologies (final costs for payers) in their analyses rather than exogenous costs, which reflect true production costs.^28^ This undermines the value of cost-effectiveness analysis that relates to the best use of resources for maximal health benefits.^29,30^ The opportunity cost would be lower societal benefits for alternative resources without maximizing health outcomes.^29,30^ This implies that cost-effectiveness analysis would not be considered an optimal method alone in HTA to influence decisions on technology adoption.

### Limitations

Although this study has been designed with a mixed model methodology, there are some limitations. First, domains and subcriteria identified from the literature included in this study come from the limited number of studies found, which might not capture the full spectrum of relevant domains for all hospitals. However, the first step in this study was to validate domains in the literature and determine if other domains should be included. This resulted in 11 additional subcriteria added to the analysis. Second, adoption criteria for new surgical technology were determined by pairwise-comparison matrices, and expert responses may, in part, reflect the Canadian health care system in which they worked. This may affect the generalizability of subcriteria in other countries with different health care systems. Additionally, numerous questions can lead to fatigue and confound some findings. Thus, questions were asked in more than 1 way (pairwise and direct ranking) to ensure that responses were reproducible. Third, prioritization and ranking needs to be a dynamic process. Domains and weights identified in this study reflect a static assessment of present surgical and hospital priorities and should be reassessed over time to stay current and accurately reflect the technology and patient needs. Fourth, more experts, representing all provinces and surgical disciplines and with a more equitable distribution of sexes would be needed for further criteria validation. Although it would be ideal if our cohort had been half male and half female, the 27% of respondents in our study who were female is not different than the distribution of surgeons in Canada.

## Conclusions

In this decision analytic modeling study of criteria prioritization to guide decision-makers for early adoption of surgical technologies, we found that not all criteria were equally weighted in importance and established weighted prioritized domains. There was a consensus among surgeons, HTA committee members, and hospital administrators on the most important decision-making domain. Although we found that clinical outcomes were the most important domain to adopt new surgical technologies, further research is needed for countries with different socioeconomic and geopolitical systems that may have different priority criteria.

## Notes

Supplement 1. **eTable 1.** Pairwise Comparison Matrix **eTable 2.** Checking for Consistency and Calculating λ **eAppendix.** Questionnaire Template Used for Study

Supplement 2.
Data Sharing Statement
