# Supplementary material for: Ranking Decision-Making Criteria for Early Adoption of Innovative Surgical Technologies
Source: JAMA Netw Open. 2023 Nov 16;6(11):e2343703. doi: 10.1001/jamanetworkopen.2023.43703 (PMC10654796; doi:10.1001/jamanetworkopen.2023.43703)
Supplement: Supplement 1. — eTable 1. Pairwise Comparison Matrix eTable 2. Checking for Consistency and Calculating λ eAppendix. Questionnaire Template Used for Study [file jamanetwopen-e2343703-s001.pdf]

Supplemental Online Content

Shoman H, Almeida ND, Tanzer M. Ranking decision-making criteria for early adoption of innovative surgical technologies. *JAMA Netw Open*. 2023;6(11):e2343703. doi:10.1001/jamanetworkopen.2023.43703

eTable 1. Pairwise Comparison Matrix

eTable 2. Checking for Consistency and Calculating λ

eAppendix. Questionnaire Template Used for Study

This supplemental material has been provided by the authors to give readers additional information about their work.

© 2023 Shoman H et al. *JAMA Network Open.*

		C1	C2	C3	C4	C5	C6	C7

Pairwise comparison matrix (Criteria comparison matrix "C") - ALL PARTICIPANTS

		**Economic**	**Hospital specific**	**Technology specific**	**Patients' / public relevance**	**Clinical outcomes**	**Policies and procedures**	**Physician specific**
R1	**Economic**	1	0.995	0.681	0.682	0.188	1.078	0.975
R2	**Hospital specific**	1.005	1	1.265	0.688	0.210	1.413	1.290
R3	**Technology specific**	1.469	0.790	1	0.544	0.200	1.449	0.986
R4	**Patients/public relevance**	1.466	1.453	1.837	1	0.320	1.641	1.396
R5	**Clinical outcomes**	5.313	4.772	5.000	3.128	1	5.018	4.862
R6	**Policies and procedures**	0.928	0.708	0.690	0.609	0.199	1	0.869
R7	**Physician specific**	1.026	0.775	1.014	0.716	0.206	1.151	1

© 2023 Shoman H et al. *JAMA Network Open.*

Checking for consistency and calculating Lambda

WEIGHT	Economic	Hospital specific	Technology specific	Patients' / public relevance	Clinical outcomes	Policies and procedures	Physician specific	Weighted Sum Values (WS)	Weights (W)	Lambda "λ" (WS/W)	LAMBDA MAX "λ-M"
**Economic**	0.083	0.099	0.063	0.092	0.081	0.081	0.085	0.583	0.083	7.038	
**Hospital specific**	0.083	0.099	0.116	0.093	0.090	0.106	0.112	0.700	0.099	7.046	
**Technology specific**	0.122	0.079	0.092	0.073	0.086	0.109	0.086	0.646	0.092	7.034	
**Patients/public relevance**	0.121	0.144	0.169	0.135	0.137	0.124	0.122	0.952	0.135	7.062	
**Clinical outcomes**	0.440	0.474	0.459	0.422	0.429	0.378	0.424	3.025	0.429	7.056	
**Policies and procedures**	0.077	0.070	0.063	0.082	0.085	0.075	0.076	0.529	0.075	7.031	
**Physician specific**	0.085	0.077	0.093	0.097	0.088	0.087	0.087	0.614	0.087	7.040	
											**7.044**

**Calculate Consistency**			
**Consistency Index**	(Lambda Max-n) / (n-1)<br>(7.044 - 7) / (7-1) =		0.007
**CR = CI/RI**	**0.006**		
**N=**	7		
**Random Index (RI)**	1.32		

© 2023 Shoman H et al. *JAMA Network Open.*

QUESTIONNAIRE TEMPLATE USED FOR STUDY:

Study Title:

Priority criteria setting for decision-making for the purchase and adoption of new surgical innovations into the Canadian Healthcare System.

Description:

Below is a list of currently used criteria for the adoption of surgical technologies in hospitals. They are in no specific order. We are interested in the criteria that you feel are important in the decision to purchase and adopt a **new surgical technology in the early adoption phase – ie. early on after its release and before the technology is commonly used**. Your input will help us in weighing which criteria are considered a priority, so as to aid surgeons, administrators and government agencies in their decisions regarding adopting a new surgical technology.

Instructions:

Kindly fill the below tables by checking the appropriate box.

Estimated time: 12 minutes

Demographic data (*Please type in your name and click on the relevant box to check it*):

**Name**	
**Highest level of Education**	☐ MD or equivalent (eg: BSc, BA)
	☐ Masters
	☐ PhD
	☐ < 10 years

© 2023 Shoman H et al. *JAMA Network Open.*

**Years of experience in your profession (cumulative)**	☐ 11 – 20 years
	☐ 21 – 30 years
	☐ > 30 years

Survey Question 1:

Using the 5-point Likert scale, please check by clicking in the box that indicates the level of importance that you put on each of the following 7 criteria categories and their sub-criteria when deciding to purchase and adopt a new surgical technology in its early adoption phase.

CRITERIA	1 (Irrelevant)	2 (Less important)	3 (Neutral)	4 (Average importance)	5 (Absolute importance)
**1- ECONOMIC**	☐	☐	☐	☐	☐
1.1- Cost	☐	☐	☐	☐	☐
1.2 – Economic impact	☐	☐	☐	☐	☐
1.3- Cost effectiveness	☐	☐	☐	☐	☐
1.4- Depreciation cost	☐	☐	☐	☐	☐
**2- HOSPITAL SPECIFIC**	☐	☐	☐	☐	☐
2.1- Feasibility of implementation	☐	☐	☐	☐	☐
2.2- Structural / management support	☐	☐	☐	☐	☐
2.3- Strategic fit	☐	☐	☐	☐	☐
2.4- Relevance	☐	☐	☐	☐	☐
2.5- Standards of care	☐	☐	☐	☐	☐
2.6- Service coordination	☐	☐	☐	☐	☐
2.7- Being an academic and clinical center for excellence	☐	☐	☐	☐	☐
**3- TECHNOLOGY SPECIFIC**	☐	☐	☐	☐	☐

© 2023 Shoman H et al. *JAMA Network Open.*

3.1- Technology simplicity	☐	☐	☐	☐	☐
3.2- Innovation	☐	☐	☐	☐	☐
3.3- Quality	☐	☐	☐	☐	☐
3.4- Real time feedback	☐	☐	☐	☐	☐
3.5- Efficiency	☐	☐	☐	☐	☐
**CRITERIA**	**1 (Irrelevant)**	**2 (Less important)**	**3 (Neutral)**	**4 (Average importance)**	**5 (Absolute importance)**
3.6- Maintenance availability	☐	☐	☐	☐	☐
3.7- Available evidence (quality)	☐	☐	☐	☐	☐
3.8- Alternatives available	☐	☐	☐	☐	☐
**4- PATIENTS' / PUBLIC RELEVANCE**	☐	☐	☐	☐	☐
4.1- Population health impact	☐	☐	☐	☐	☐
4.2- Human responses / patient experience	☐	☐	☐	☐	☐
4.3- Publicity and awareness	☐	☐	☐	☐	☐
4.4- Access	☐	☐	☐	☐	☐
4.5- Social and demographic	☐	☐	☐	☐	☐
**5- CLINICAL OUTCOMES**	☐	☐	☐	☐	☐
5.1- Safety	☐	☐	☐	☐	☐
5.2- Efficacy	☐	☐	☐	☐	☐
5.3- Effectiveness	☐	☐	☐	☐	☐
5.4- Prevention of adverse effects	☐	☐	☐	☐	☐
5.5-Evidence of peer reviewed assessments	☐	☐	☐	☐	☐
5.6- Disease burden	☐	☐	☐	☐	☐
**6- POLICIES AND PROCEDURES**	☐	☐	☐	☐	☐

© 2023 Shoman H et al. *JAMA Network Open.*

6.1- Ethical	☐	☐	☐	☐	☐
6.2- Legislative	☐	☐	☐	☐	☐
6.3- Environmental	☐	☐	☐	☐	☐
6.4- Sustainability	☐	☐	☐	☐	☐
6.5- Political	☐	☐	☐	☐	☐
6.6- Appeals	☐	☐	☐	☐	☐
**CRITERIA**	**1 (Irrelevant)**	**2 (Less important)**	**3 (Neutral)**	**4 (Average importance)**	**5 (Absolute importance)**
6.7- Enforcement	☐	☐	☐	☐	☐
6.8- Certification of technology (Health Canada – ISO)	☐	☐	☐	☐	☐
**7- PHYSICIAN SPECIFIC**	☐	☐	☐	☐	☐
7.1- Sense of security	☐	☐	☐	☐	☐
7.2- Flexibility of usage	☐	☐	☐	☐	☐
7.3- Innovation champions	☐	☐	☐	☐	☐
7.4- Training	☐	☐	☐	☐	☐
7.5- Percentage of utilization	☐	☐	☐	☐	☐
7.6- Availability of the technology	☐	☐	☐	☐	☐

© 2023 Shoman H et al. *JAMA Network Open.*

Survey Question 2:

For each line below, compare criteria A vs criteria B. Please determine which criteria (criteria A vs criteria B) is more important and rate them using 1-9 by checking the box.

E.g.: I believe the **shape** of the technology is *absolutely* *more important* than its **color** in influencing my decision. → Check box under number 9 closer to the **Criteria shape**

The criteria are defined by the same sub-criteria that are listed in Question 1.

MAIN CATEGORIES																		
**Criteria A**	9 Absolute importance	8	7 Very strong importance	6	5 Strong importance	4	3 Moderate importance	2	**EQUAL** 1 Equal importance	2	3 Moderate importance	4	5 Strong importance	6	7 Very strong importance	8	9 Absolute importance	**Criteria B**
Color	☐	☐	☐	☐	☐	☐	☐	☐	☐	☐	☐	☐	☐	☐	☐	☐	☒	Shape
Economic	☐	☐	☐	☐	☐	☐	☐	☐	☐	☐	☐	☐	☐	☐	☐	☐	☐	Hospital specific
Economic	☐	☐	☐	☐	☐	☐	☐	☐	☐	☐	☐	☐	☐	☐	☐	☐	☐	Technology specific
Economic	☐	☐	☐	☐	☐	☐	☐	☐	☐	☐	☐	☐	☐	☐	☐	☐	☐	Patients’ / public relevance
Economic	☐	☐	☐	☐	☐	☐	☐	☐	☐	☐	☐	☐	☐	☐	☐	☐	☐	Clinical outcomes
Economic	☐	☐	☐	☐	☐	☐	☐	☐	☐	☐	☐	☐	☐	☐	☐	☐	☐	Policies and procedures
Economic	☐	☐	☐	☐	☐	☐	☐	☐	☐	☐	☐	☐	☐	☐	☐	☐	☐	Physician specific
Hospital specific	☐	☐	☐	☐	☐	☐	☐	☐	☐	☐	☐	☐	☐	☐	☐	☐	☐	Technology specific
Hospital specific	☐	☐	☐	☐	☐	☐	☐	☐	☐	☐	☐	☐	☐	☐	☐	☐	☐	Patients’ / public relevance

© 2023 Shoman H et al. *JAMA Network Open.*

Hospital specific	☐	☐	☐	☐	☐	☐	☐	☐	☐	☐	☐	☐	☐	☐	☐	☐	☐	Clinical outcomes
**Criteria A**	**9 Absolute importance**	**8**	**7 Very strong importance**	**6**	**5 Strong importance**	**4**	**3 Moderate importance**	**2**	**1 Equal importance**	**2**	**3 Moderate importance**	**4**	**5 Strong importance**	**6**	**7 Very strong importance**	**8**	**9 Absolute importance**	**Criteria B**
Hospital specific	☐	☐	☐	☐	☐	☐	☐	☐	☐	☐	☐	☐	☐	☐	☐	☐	☐	Policies and procedures
Hospital specific	☐	☐	☐	☐	☐	☐	☐	☐	☐	☐	☐	☐	☐	☐	☐	☐	☐	Physician specific
Technology specific	☐	☐	☐	☐	☐	☐	☐	☐	☐	☐	☐	☐	☐	☐	☐	☐	☐	Patients' / public relevance
Technology specific	☐	☐	☐	☐	☐	☐	☐	☐	☐	☐	☐	☐	☐	☐	☐	☐	☐	Clinical outcomes
Technology specific	☐	☐	☐	☐	☐	☐	☐	☐	☐	☐	☐	☐	☐	☐	☐	☐	☐	Policies and procedures
Technology specific	☐	☐	☐	☐	☐	☐	☐	☐	☐	☐	☐	☐	☐	☐	☐	☐	☐	Physician specific
Patients' / public relevance	☐	☐	☐	☐	☐	☐	☐	☐	☐	☐	☐	☐	☐	☐	☐	☐	☐	Clinical outcomes
Patients' / public relevance	☐	☐	☐	☐	☐	☐	☐	☐	☐	☐	☐	☐	☐	☐	☐	☐	☐	Policies and procedures
Patients' / public relevance	☐	☐	☐	☐	☐	☐	☐	☐	☐	☐	☐	☐	☐	☐	☐	☐	☐	Physician specific
Clinical outcomes	☐	☐	☐	☐	☐	☐	☐	☐	☐	☐	☐	☐	☐	☐	☐	☐	☐	Policies and procedures
Clinical outcomes	☐	☐	☐	☐	☐	☐	☐	☐	☐	☐	☐	☐	☐	☐	☐	☐	☐	Physician specific
Policies and procedures	☐	☐	☐	☐	☐	☐	☐	☐	☐	☐	☐	☐	☐	☐	☐	☐	☐	Physician specific

© 2023 Shoman H et al. *JAMA Network Open.*

Thank you for your time and expertise. It is very appreciated!

**For any questions, contact: Haitham Shoman haitham.shoman@mail.mcgill.ca*

© 2023 Shoman H et al. *JAMA Network Open.*
