# Supplementary material for: Ranking Decision-Making Criteria for Early Adoption of Innovative Surgical Technologies
Source: JAMA Netw Open. 2023 Nov 16;6(11):e2343703. doi: 10.1001/jamanetworkopen.2023.43703 (PMC10654796; doi:10.1001/jamanetworkopen.2023.43703)
Supplement: Supplement 2. — Data Sharing Statement [file jamanetwopen-e2343703-s002.pdf]

Data Sharing Statement

Shoman. Ranking Decision-Making Criteria for Early Adoption of Innovative Surgical Technologies. *JAMA Netw Open*. Published online November 16, 2023. doi:10.1001/jamanetworkopen.2023.43703

Data
Data available: No

Additional Information
Explanation for why data not available: We do not have any patient data in our research.
